# Evaluation of CareStart™ malaria Pf/Pv combo test for *Plasmodium falciparum* and *Plasmodium vivax* malaria diagnosis in Butajira area, south-central Ethiopia

**DOI:** 10.1186/1475-2875-12-218

**Published:** 2013-06-27

**Authors:** Adugna Woyessa, Wakgari Deressa, Ahmed Ali, Bernt Lindtjørn

**Affiliations:** 1 Ethiopian Health & Nutrition Research Institute, P. O. Box 1242, Addis Ababa, Ethiopia; 2 School of Public Health, College of Health Sciences, Addis Ababa University, P. O. Box 9086, Addis Ababa, Ethiopia; 3 Centre for International Health, University of Bergen, Bergen, Norway

**Keywords:** CareStart^TM^ RDT, Microscopy, Precision, Season, Highland, Butajira, Ethiopia

## Abstract

Malaria is a major public health problem in Ethiopia. *Plasmodium falciparum* and *Plasmodium vivax* co-exist and malaria rapid diagnostic test (RDTs) is vital in rendering parasite-confirmed treatment especially in areas where microscopy from 2008 to 2010 is not available. CareStart^TM^ Malaria Pf/Pv combo test was evaluated compared to microscopy in Butajira area, south-central Ethiopia. This RDT detects histidine-rich protein-2 (HRP2) found in *P. falciparum*, and *Plasmodium* enzyme lactate dehydrogenase (pLDH) for diagnosis of *P. vivax*. The standard for the reporting of diagnostic accuracy studies was complied. Among 2,394 participants enrolled, 10.9% (n=87) were *Plasmodium* infected (household survey) and 24.5% (n=392) health facility-based using microscopy. In the household surveys, the highest positivity was caused by *P. vivax* (83.9%, n=73), *P. falciparum* (15.0%, n=13), and the rest due to mixed infections of both (1.1%, n=1). In health facility, *P. vivax* caused 78.6% (n=308), *P. falciparum* caused 20.4% (n=80), and the rest caused by mixed infections 1.0% (n=4). RDT missed 9.1% (n=8) in household and 4.3% (n=17) in health facility-based surveys among *Plasmodium* positive confirmed by microscopy while 3.3% (n=24) in household and 17.2% (n=208) in health facility-based surveys were detected false positive. RDT showed agreement with microscopy in detecting 79 positives in household surveys (n=796) and 375 positives in health centre survey (n=1,598).

RDT performance varied in both survey settings, lowest PPV (64.3%) for *Plasmodium* and *P. falciparum* (77.2%) in health centres; and *Plasmodium* (76.7%) and *P. falciparum* (87.5%) in household surveys. NPV was low in *P. vivax* in health centres (77.2%) and household (87.5%) surveys. Seasonally varying RDT precision of as low as 14.3% PPV (Dec. 2009), and 38.5% NPV (Nov. 2008) in health centre surveys; and 40-63.6% PPV was observed in household surveys. But the influence of age and parasite density on RDT performance was not ascertained. Establishing quality control of malaria RDT in the health system in areas with low endemic and where *P. falciparum* and *P. vivax* co-exist is recommendable. CareStart^TM^ RDT might be employed for epidemiological studies that require interpreting the results cautiously. Future RDT field evaluation against microscopy should be PCR corrected.

## Background

In Ethiopia, approximately 68% of the recently estimated 92 million total population, about 63 million people, is at risk of malaria infection [1,2]. Typically malaria is seasonal and unstable [3], and consequently epidemics of different magnitude were common in the past [3-5]. Even though the occurrence of all human malaria parasites is reported, *Plasmodium falciparum* and *Plasmodium vivax* are epidemiologically the most important malaria parasites in the country [4]. *Plasmodium falciparum* has been the major causes of high case fatality. Since 2005, the National Malaria Control Programme intensified the deployment of key malaria interventions including artemisinin-combination therapy (ACT), malaria rapid diagnostic tests (RDTs), and vector control measures [6]. Thus, in recent years, malaria burden decreased [7,8], and large-scale epidemics were absent [9].

Malaria RDTs were introduced as one of the diagnostic methods following the revision of malaria diagnosis and treatment guideline in 2004 [10]. The principle of a RDT is to capture malaria antigen from peripheral blood flowing across a membrane containing specific anti-malaria antibodies. Thus, there are different types of RDTs depending on the antigen they target: those targeted to histidine-rich protein-2 (HRP-2) only detect *P. falciparum*, those which target the parasite enzyme lactate dehydrogenase (LDH) and aldolase that can detect non- falciparum from mixed infection. LDH test uses either monoclonal antibodies which react with LDH of all species including *P. falciparum* (or known as Pan-LDH), or antibodies specific for *P. falciparum* LDH [11-13]. RDTs can also detect *P.vivax*-specific LDH.

RDTs for detection of only *P. falciparum* were recommended [10], and have been used at health posts in Ethiopia since 2005 [14,15]. However, RDTs that detect HRP-2 have got some practical limitations and guide treatment [13,16]. Following the intensive malaria interventions since 2005, the dominance of *P. vivax* was documented in some highland areas [17,18]. At community level malaria diagnosis and treatment using RDTs target *P. falciparum,* and consequently *P. vivax* may be misdiagnosed as malaria negative and treated based on clinical grounds. Moreover, World Health Organization (WHO) recommends that malaria case management be based on parasite-based diagnosis [19]. Thus, the introduction of RDTs targeted to detect both *P. falciparum* and *P. vivax* is helpful in improving the early diagnosis and treatment of malaria at peripheral areas. CareStart™ Malaria Pf/Pv combo test was among multi-species detecting RDT according to WHO product testing [20]. Various field evaluations of CareStart™ Malaria Pf/Pv combo test were underway since 2007 in different parts of Ethiopia [21-25]. However, in general, malaria diagnostics have performed differently across eco-epidemiological settings, leading to varying estimates of agreement between tests [26-28].

There is paucity of information regarding the precision of malaria RDTs in the low-endemic highland zones of Ethiopia. Therefore, the present study was aimed at evaluating the performance of CareStart™ at health centres and concurrent surveys in highland areas of south-central Ethiopia.

## Methods

### Study area and population

This study was conducted in Butajira area, situated 130 km south of Addis Ababa, in six of ten *kebele*s (the smallest administrative unit) part of the Demographic Surveillance site (DSS) of Butajira Rural Health Programme (BRHP) [29], and administratively located in Meskan District, Gurage Zone, Ethiopia. Butajira Town is the base of the BRHP-DSS, which is located along Addis Ababa-Hosaena highway and 50 km West of Ziway Town, adjacent to Ziway Lake in the Rift Valley. The study area is situated in intermediate highland zone with elevation of between 1,800 and 2,300 meters above sea level (masl). Moreover, the study area was part of the Ethiopian Malaria Prediction System project [30]. The detailed description of the study area was presented in another recent study [31].

There were 58,335 people living in the BRHP-DSS in 2008 and half (50.1%, n = 29,243) of the population were female. Of those 26,834 (46%) people lived in the study area. Malaria is endemic in the area with frequent outbreak that caused significant cases and deaths as documented previously [32]. The local population get in-patient services at Butajira Zonal Hospital (a public-owned), which is located in Butajira Town, and Mercy Hospital (a non-governmental organization situated about 10 km outside the town). In addition, out-patient services are obtained from Butajira, Enseno, and Hamus Gebeya health centres as well as at private clinics. Moreover, each study *kebele* has a health post. Drug venders are also available in Butajira Town.

This study was conducted while the National Malaria Control Program of Ethiopia has initiated various local academic and research institutions for field evaluation of RDTs that could detect multi-species and replace RDTs that only detect *P. falciparum*. Thus, CareStart^TM^ Malaria Pf/Pv combo test was among the options for the field evaluation before introducing to malaria control program of the country.

### Survey design and sampling

A prospective household and health facility-based surveys were conducted to assess the performance of CareStart™ Malaria Pf/Pv combo test in reportedly febrile cases between 2008 and 2010. Six rural kebeles such as Hobe, Bati Lejano, Dirama, Shershera Bido, Yeteker and Wurib were randomly selected. A total of 750 households were selected randomly from those kebeles, using probability proportion to size (PPS) sampling. Individuals with the history of fever in the previous 48 hours of the survey period were included in both household and health facility-based surveys. Febrile cases visiting outpatient department (OPD) suspected of malaria cases at Butajira and Enseno health centres were recruited. In general, this study complied with Standards for Reporting of Diagnostic Accuracy guidelines (STARD) to improve the quality of reporting [33].

**Figure 1:**
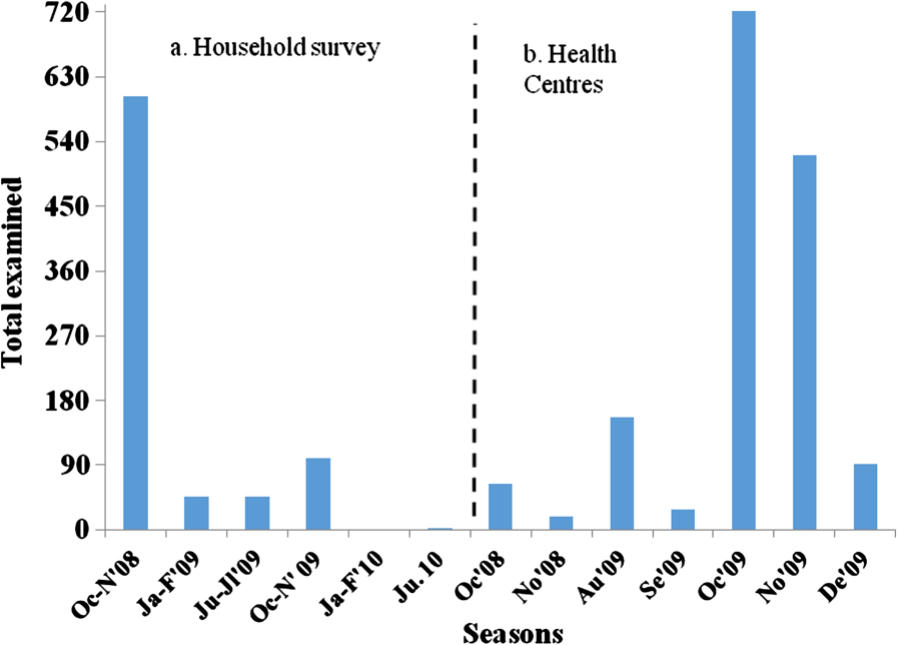
Number of participants varying among survey seasons, Butajira area, Ethiopia, 2008-2010.

Six cross-sectional surveys were performed on the same households for two consecutive years. The surveys were conducted in October-November 2008 (a month after the main rainy season), January-February 2009 (dry season), June-July 2009 (main rainy season), October-November 2009, January-February 2010, and June 2010 in the household surveys. The health facility-based survey was done during October 2008, November 2008, August 2009, September 2009, October 2009, November 2009, and December 2009 (Figure 1). The health facility-based survey was done at Butajira Health Centre (≥ 2, 000 masl) and Enseno Health Centre (< 2,000 masl). Individuals with a history of fever in the previous 48 hours were enrolled. But severely-ill people were excluded.

### Data collection

Data collection was done using pre-tested structured questionnaire and format prepared for this purpose. Pre-testing of the data collection tool was performed in an adjacent *kebele* but outside the study area. Intensive training of data collectors and supervisors was conducted. All data collectors were supplied with a training manual and field guide. Data collectors and supervisors for both the household and health facility-based surveys were recruited from local public health facilities. BRHP-DSS enumerators and supervisors did household interview in the baseline household survey, and guided and facilitated the data collection in the follow up of household surveys. A coordinator of the Butajira base facilitated recruitment of data collectors and supervised the data collection with the principal investigator (AW).

The health facility-based survey was organized as a research team of four health personnel assigned from the same health centre. Overall, the study was supervised by the principal investigator (AW), a laboratory technician, and two OPD staffs were the member of the team. A health centre head was responsible for coordination of the survey. Those OPD staffs each assigned to screen malaria suspected febrile cases at pediatric and adult OPD separately. During the data collection, participants were requested for blood specimen to be screened for malaria parasite using CareStart™ Malaria Pf/Pv combo test (Access Bio, Inc., New Jersey, USA; LOT H28: V, Expiry July 2010). In addition, thin and thick film was prepared immediately for microscopic examination. CareStart™ RDTs are individually packaged with alcohol swab, lancet, capillary tube, buffer, and test device.

Both blood slides and the RDT devices were given identical code for later comparison of results. Specimen collection and reporting result for the malaria RDT was performed using the recommended procedures by the company [34]. First, 5 μl of blood specimen was added into sample well of the test device using a capillary supplied with the kit for this purpose. Second, two drops of assay buffer was added into the buffer well. The results of CareStart™ were declared within 20 minutes, while thin and thick films were kept for later examination using microscopy. The RDT result was recorded along a registry book using the participant code.

Then, thin and thick films were prepared and Giemsa-stained for comparison using light microscopy. Blood specimen processing, examination, and reporting of results were performed using standard guidelines [35]. In brief, the smears were air dried, placed in slide boxes, and examined by a trained microscopist at the field laboratory in Butajira. Thin films were fixed with methanol, and both thin and thick films were stained with 3% Giemsa stain for 30 min. Microscopic examination was done at 1,000× magnification. During the microscopic examination, a slide was regarded as negative after 100 fields had been examined without finding any parasites. When slides were *Plasmodium* positive, asexual parasite count was performed using standard procedures of light microscopy as recommended by World Health Organization [35]. The number of asexual parasites per 200 white blood cells (WBCs), or 500 WBCs for low density infections, were used to compute the number of asexual parasites per μl of blood, assuming a standard count of 8,000 WBCs per μl of blood [35].

### CareStart™ Malaria Pf/Pv combo RDT and interpretation of the test

CareStart™ Malaria Pf/Pv combo test contains a membrane strip, which is pre-coated with two monoclonal antibodies as two separate lines across a test strip. One monoclonal antibody (test line 2) is specific to pLDH of *P. vivax*. Other lines (test line 1) is printed with a monoclonal antibody specific to HRP2 of *P. falciparum*, and the control area (line 3). So, the CareStart™ Malaria Pf/Pv combo test is designed for the differential diagnosis between *P. vivax* and *P. falciparum*[34]. The following five options including the presence of: i) two bands (one band in the control area and another band in the “1” area) indicates a positive result for *P. falciparum*; ii) two colour bands (one band in the control area and another band in the “2” area) indicates a positive result for *P. vivax*; iii) three colour bands (bands in the control area, “1” area and “2” area) indicates a positive result for mixed infection for *P. falciparum* and *P. vivax*; iv) only one band in the control area within the result window indicates a negative result; and v) the test is invalid if the line in the control area does not appear were used in the interpretation of RDT results [34]. In addition, the intensity of bands was carefully recorded for the test positives using guidelines for interpretation of RDT results available from the manufacturing company.

### Comparison with light microscopy and quality control

A malaria microscopist temporarily stationed at BRHP-DSS [29], did the microscopic examination using standard procedure [35]. In the household surveys, a single slide was prepared from each individual. Thin and thick blood films from were dried and daily transported to the temporary laboratory for staining and parasite investigation. Similarly, the slides from health centres were processed and investigated for malaria parasite at the same laboratory. The malaria microscopist was blinded to malaria RDT results. Similarly, health professionals were blinded to microscopy results. Febrile cases requested for blood film to be checked for malaria using CareStart™. Study participants were sent to laboratory and duplicate slides with thin and thick films were produced simultaneously. One of the slides was used for the routine microscopic examination and results dispatched accordingly. Another slide was processed and regularly sent to the temporary laboratory at BRHP-DSS.

In order to assure quality of the microscopic examinations, in both surveys, the entire positive and 10% of the negative slides were sent to senior malaria microscopist and re-examined at Adama Malaria Control Laboratory, Adama Town, Ethiopia. In the household surveys, seven of 2,094 slides (0.3%) showed discordant results. A third reader, blinded to the previous results, re-examined the seven discrepant slides (six vivax malaria and one negative). Similar procedure was followed for the survey held at health centres. Each reader was blinded to the RDT results and others’ slide reading results. To ensure maximum participation, households with absentees were re-visited once.

### Ethical consideration

This study obtained ethical approval from Institutional Review Board of the College of Health Sciences of the Addis Ababa University, and from the Ministry of Science and Technology of Ethiopia. Local health authorities at all levels also gave permission to conduct the study. Individual informed consent was obtained from adults, and from the parents or guardians of children aged less than 18 years. In addition, minors gave verbal assent. All study participants including minors had the right to refuse. Blood specimens were collected aseptically using an alcohol swab and disposable blood lancets by trained staff. All people found to be malaria positive during the survey were treated directly according to the national guideline [10].

Febrile patients were screened for malaria parasites on the spot using the RDT, and blood slides were also collected for the survey to investigate the nature of the malaria parasite using light microscopy. Malaria positive cases detected using malaria RDTs were treated immediately. Moreover, those found to be malaria positive on microscopy were also treated. *Plasmodium vivax* positives were treated with chloroquine, 25 mg/kg for three days (10 mg base per kg on days 1 and 2, and 5 mg base per kg on day 3). Artemether-lumefantrine (20 mg artemether plus 120 mg lumefantrine in a fixed dose combination) was administered, based on body weight, two times a day for three days to *Plasmodium falciparum*-positive patients [10]. In addition, Coartem® and chloroquine stocks were kept at health facilities in the study area for treatment of cases that occurred between surveys.

### Data management and analysis

Data entry and cleaning was done using Epi Info version 6 (Centres for Disease Control and Prevention, Atlanta, Georgia, USA) and exported to SPSS. Data analysis was performed using IBM SPSS Statistics 20. Sensitivity, specificity, positive and negative predictive values of the test was computed with 95% confidence interval (CI) by taking malaria microscopy as reference.

The kappa statistic or agreement between the test and microscopy was calculated using data observed and probability of expected as recommended [36]. A kappa value of 1 indicates perfect agreement, whereas a kappa of 0 indicates agreement equivalent to chance. The other kappa values of 0.81-0.99 (almost perfect), 0.61-0.80 (substantial), 0.41-0.60 (moderate), 0.21-0.40 (fair), 0.01-0.20 (slight), and <0 (less than chance) agreements were used for interpretation [36]. The evaluation of the performance of the test was based on requirement that RDTs must achieve, which is expected to be ≥ 95% sensitivity and >90% specificity to be useful diagnostic [37]. The results from January-February 2010 and June 2010 surveys was excluded from analysis of age and seasonal variation of RDT performance because of finding null and a single malaria positive, respectively.

## Results

### Characteristics of study participants

The evaluation of CareStart^TM^ performance was based on self-reported 2,394 febrile cases from public health facilities (66.8%, n = 1,598) and household surveys (33.2%, n = 796). The participants’ mean (±SD) age was 13.9 ± 13.0 (from one month to 70 years), and median 10 years in health centre surveys, and 19.7 ± 18.2 (from one month to 99 years) with median 13 years in household surveys. More participants were recruited from Enseno Health Centre (73.7%, 1,178 of 1,598) than from Butajira Health Centre (26.3%, 420 of 1,598) in health facility-based surveys. A total of 31 infants (six from household and 25 from health centre surveys) were suspected for malaria screened (Data not shown).

Seasonal variation of malaria suspected cases observed in both survey settings, which was higher during October-November 2008 in household surveys, and October 2009 and November 2009 at health centres (Figure 1).

### Microscopy results

**Figure 2:**
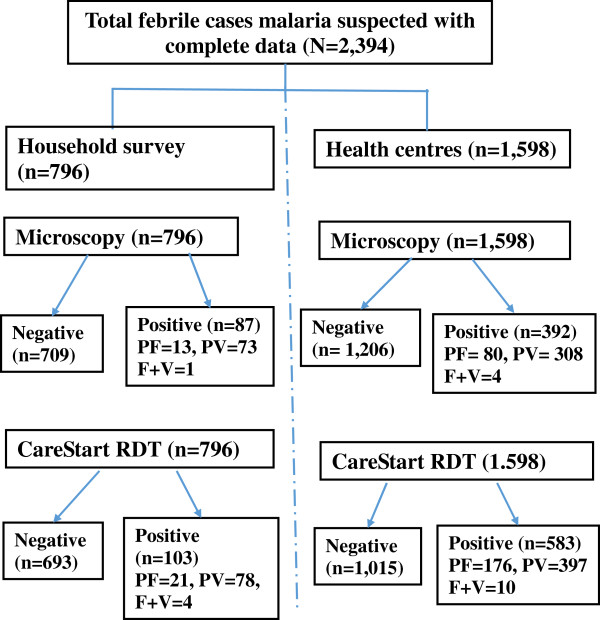
Flow chart of microscopy and CareStartTM Malaria RDT results, Butajira area, Ethiopia, 2008-2010.

The microscopic results showed that 10.9% (n = 87) of people examined were *Plasmodium* positive in the household surveys. Of those, 83.9% (n = 73) were caused by *P. vivax*, 15.0% (n = 13) were due to *P. falciparum*, and the rest 1.1% (n = 1) were mixed infections due to both vivax and falciparum malaria. Similarly, 24.5% (n = 392) of people were malaria infected among febrile cases visited health centres. Among them, 78.6% (n = 308) were *P. vivax*, 20.4% (n = 80) were *P. falciparum*, and the rest 1.0% (n = 4) were mixed infections due to both vivax and falciparum malaria (Figure 2). Eight of 25 (32%) children below six months were diagnosed as *P. vivax* infected.

Microscopy and RDT showed agreement for 79 positives in household and 375 positives in health centre survey results. RDT missed 9.1% (n = 8) in household and 4.3% (n = 17) in health facility-based surveys among *Plasmodium* positive confirmed by microscopy. Similarly, 3.3% (n = 24) in household and 17.2% (n = 208) in health facility-based surveys were detected false positive. No invalid test results were observed (Figure 2).

*Plasmodium* infection varied among different age groups, and males were more infected than females in both survey settings. Higher proportion of the malaria infection occurred during peak malaria transmission in both survey settings. About 67% of the total positives cases were screened during October-November 2009 and about 20% during October-November 2008 in household surveys. Moreover, above half of the positives during October 2009 and one-fifths during August and November 2009 each, in the health facility-based surveys (Table 1).

**Table 1:** Characteristics of study participants in household and health facility-based surveys, Butajira area, Ethiopia, 2008-2010

**Factors**	**Household survey (N = 796), n%**	**Microscopy positive (n = 87), n(%)**	**Health centres (N = 1,598), n (%)**	**Microscopy positive (n = 392), n(%)**
**Age groups**				
<5	178 (22.4)	27 (31.0)	495 (31.0)	117 (29.8)
5-9	150 (18.8)	28 (32.2)	293 (18.0)	84 (21.4)
10-14	86 (10.8)	10 (11.5)	179 (11.2)	56 (14.3)
≥15	382 (48.0)	22 (25.3)	63 (39.5)	135 (34.4)
**Gender**				
Male	382 (48.0)	48 (55.2)	754 (47.2)	203 (51.8)
Female	414 (52.0)	39 (44.8)	844 (52.8)	189 (48.2)
**Seasons**				
Oct.-Nov.2008	603 (75.7)	17 (19.5)		
Oct. 2008			63 (3.9)	9 (2.3)
Nov.2008			18 (1.1)	12 (3.1)
Jan.-Feb.2009	46 (5.8)	2 (2.3)		
Jun.-Jul. 2009	46 (5.8)	9 (10.3)		
Aug. 2009			156 (9.8)	76 (19.4)
Sept. 2009			28 (1.8)	13 (3.3)
Oct.-Nov.2009	100 (12.6)	58 (66.7)		
Oct.2009			721 (45.1)	201 (51.3)
Nov.2009			521 (32.6)	78 (19.9)
Dec.2009			91 (5.7)	3 (0.8)
Jan.-Feb.2010	0			
Jun.2010	1 (0.1)	1 (1.0)		

### RDT compared to microscopy

Table 2 presents the sensitivity, specificity, positive and negative predictive values of CareStart^TM^ RDT compared to microscopy in both surveys. CareStart^TM^ RDT showed low sensitivity in overall *Plasmodium* (90.8%) and *P. falciparum* (87.5%) in household survey; and in *P. vivax* (92.8%) in health facility surveys. Similarly, low specificity observed in overall *Plasmodium* (82.7%), and *P. falciparum* (92.8%) in health facility surveys, and in *P. vivax* (87.5%) in household surveys.

**Table 2:** Performance of CareStart^TM^ Malaria Pf/Pv combo test in household and health facility-based surveys, Butajira area, Ethiopia, Oct.2008-Jun. 2010

**Setting/indicators**	**Sensitivity**	**Specificity**	**PPV**	**NPV**	***Kappa***
Health Centres					
All *Plasmodium*	95.7 (93.2-97.3)	82.7 (80.5-84.8)	64.3 (60.3-68.1)	98.3 (97.3-99.0)	0.69
P. falciparum*	95.9 (88.7-98.6)	92.8 (89.3-95.2)	77.2 (67.6-84.5)	98.9 (96.8-99.6)	0.81
P. vivax*	92.8 (89.3-95.2)	95.9 (88.7-98.6)	98.9 (96.8-99.6)	77.2 (67.6-84.5)	0.81
Household surveys					
All *Plasmodium*	90.8 (82.9-95.3)	96.6 (95.0-97.7)	76.7 (67.7-83.8)	98.8 (97.7-99.4)	0.81
P. falciparum*	87.5 (52.9-97.8)	98.5 (91.9-99.7)	87.5 (52.9-97.8)	98.5 (91.9-99.7)	0.86
P. vivax*	98.5 (91.9-99.7)	87.5 (52.9-97.8)	98.5 (91.9-99.7)	87.5 (52.9-97.8)	0.86

*Mixed infections excluded, PPV = Positive predictive value, NPV = Negative predictive value.

Moreover, lowest PPV was determined in overall *Plasmodium* (64.3%) and *P. falciparum* (77.2%) in health facility; and overall *Plasmodium* (76.7%) and *P. falciparum* (87.5%) in household surveys. Negative predictive value of the test was good in both overall *Plasmodium* and *P. falciparum*. However, lowest NPV was found in *P. vivax* in both health facility (77.2%) and household (87.5%) surveys. The test and microscopy showed almost perfect agreement for household and health centres surveys (Table 2).

### Factors affecting RDT performance

**Figure 3:**
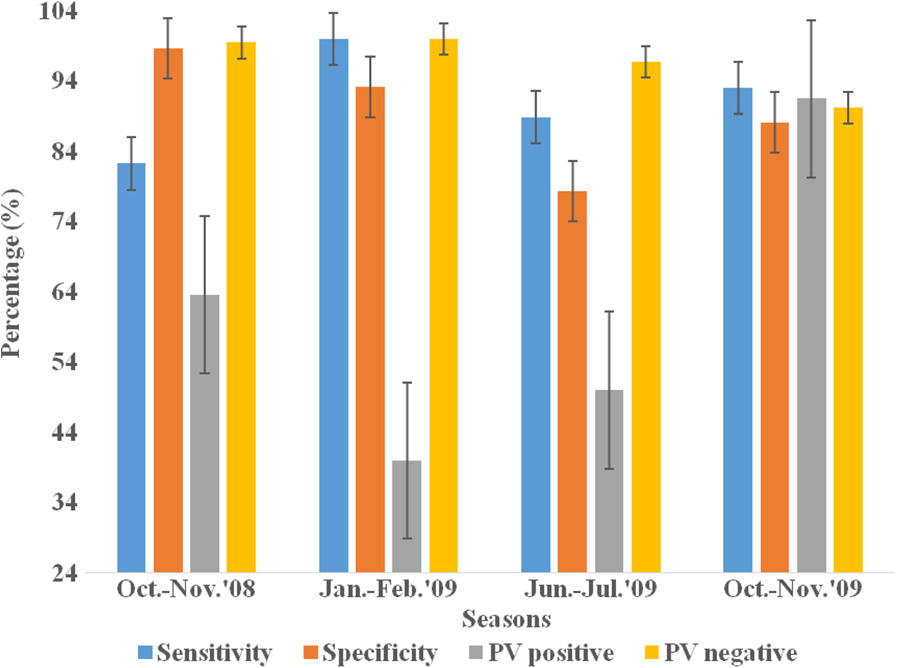
Sensitivity, specificity, predictive value positive and negative of CareStartTM Malaria RDT in different seasons in the household surveys, Butajira area, Ethiopia, Oct.2008-Nov. 2009.

**Figure 4:**
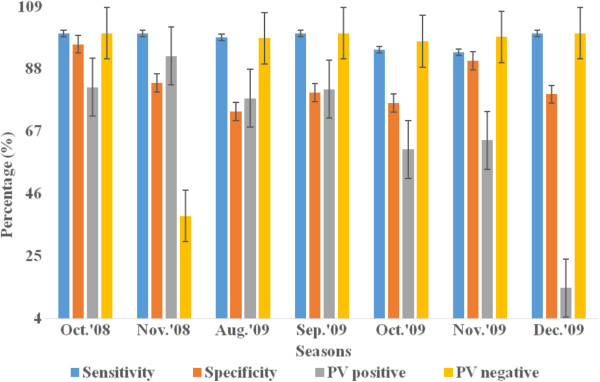
Sensitivity, specificity, predictive value positive and negative of CareStartTM Malaria RDT in different seasons in health facility surveys, Butajira area, Ethiopia, Oct.2008-Dec. 2009.

**Figure 5:**
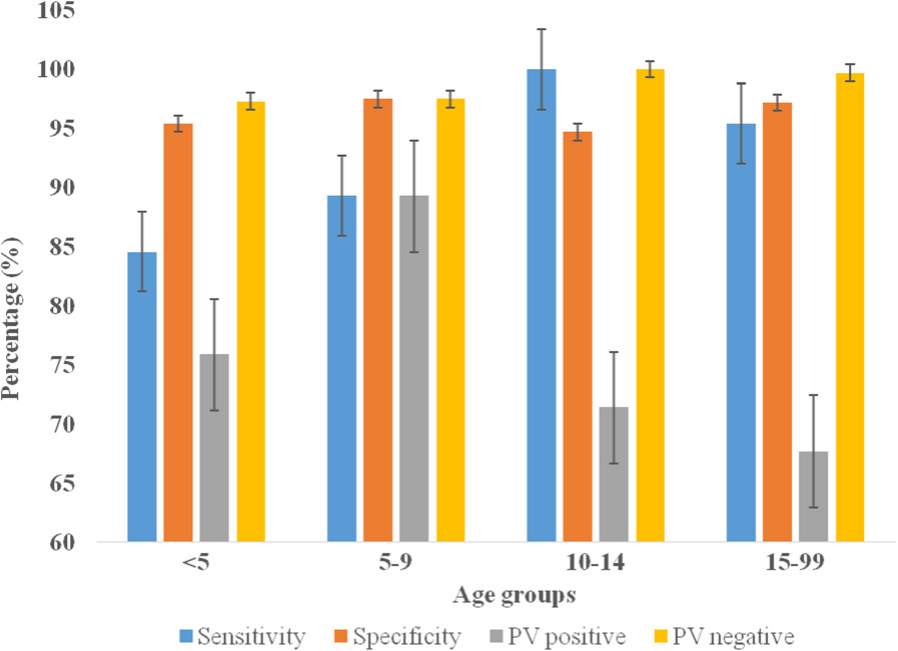
Sensitivity, specificity, predictive value positive and negative of CareStartTM Malaria RDT in different age groups in household surveys, Butajira area, Ethiopia, Oct.2008-Nov. 2010.

**Figure 6:**
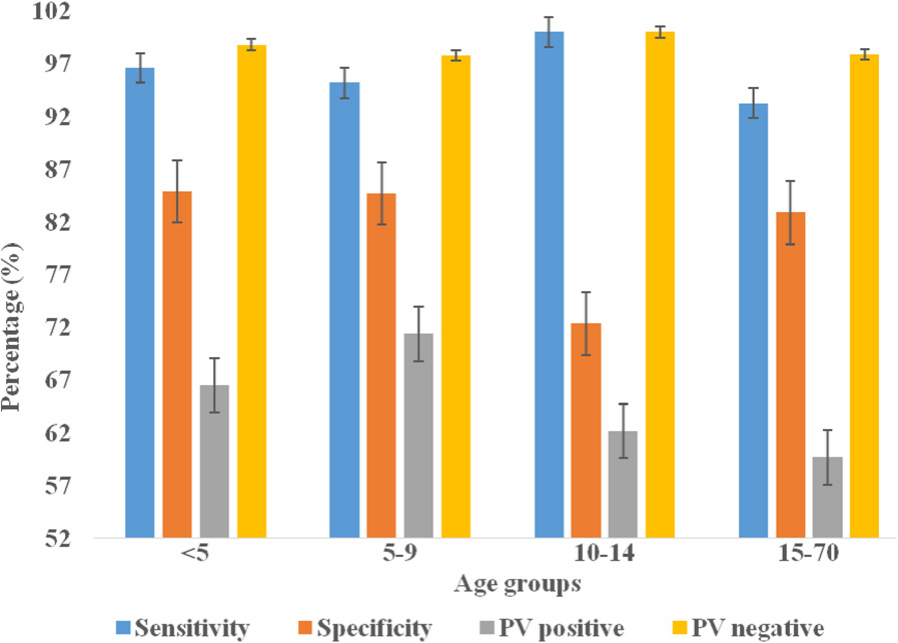
Sensitivity, specificity, predictive value positive and negative of CareStartTM Malaria RDT in different age groups in health facility surveys, Butajira area, Ethiopia, Oct.2008-Dec. 2009.

The relationship of RDT precision and survey seasons, age and parasite density in all survey settings is presented in Figures 3, 4, 5 and 6 below. Figure 3 presents varying RDT performance among different seasons in household surveys. Sensitivity varied between 82.3% and 100%, and specificity between 78.4% and 98.6%. Low sensitivity (82.3) was observed during October-November 2008 and low specificity (78.4%) during June-July 2009. The lowest PPV of 63.6% (October-November 2008), 40.0% (January-February 2009), and 50.0% (June-July 2009) was obtained; and it appears corresponding with malaria infection of 19.5%, 2.3% and 10.3%, respectively. Over all RDT precision was found to be better during October-November 2009 (Figure 3). Although a recent study reported overall low malaria prevalence (0.9%), higher malaria prevalence during October-November 2009 [31]. In health facility surveys, the sensitivity was between 93.6% and 100%. Good sensitivity of from 98.7% to 100% were observed, except the relatively low sensitivity during October 2009 (94.5%) and November 2009 (93.6%). Specificity was (73.6-96.3%) while substantial differences exist in PPV and NPV. Low PPV of 60.9-92.3% in all seasons, and the least (14.3%) in December 2009. Highest NPV (97.3-100%) was obtained, except the lowest (38.5%) was found in November 2008, which overlapped with a few malaria suspected cases (n = 18) were screened (Figure 4).

Precision of RDT in different age groups using household surveys is presented in Figure 5. Low sensitivity was seen in <5 aged (84.6%) and 5-9 aged (89.5%) children compared to older children, 10-14 years (100%) and ≥15 years (95.4%). However, good specificity with almost similar precision (94.7-97.5%) was revealed in different age categories; and low PPV (67.7-89.3%), and highest NPV (97.3-100%) was determined. In health facility-based surveys, RDT demonstrated persistently good sensitivity (95.2-100%) in children aged between <5 and 10-14 years, except 93.3% in ≥15 years. Low specificity (72.4%-84.9%) and PPV (59.7-71.4) was observed in all age groups, extremely low PPV in <5 and ≥15 years (Figure 6). NPV was the highest throughout the survey in both survey settings.

In health facilities, 4.5% (n = 10) of microscopy confirmed *P. vivax,* and 5.9% (n = 5) of *P. falciparum* with asexual parasite count of ≥ 500 p/μl were not detected by CareStart™ RDT. Moreover, the RDT failed detecting 10.3% (n = 7) of vivax malaria, five of those with ≥ 500 p/μl and the rest two with <200 p/μl in household survey. Only a single (1 of 13) falciparum case was missed by RDT in household survey (Table 3). Sixteen of 87 (18.4%) positives were reportedly sick of malaria and half of those took antimalarial treatment. Most of the cases were due to *P. vivax* (n = 15) (data not shown)*.*

**Table 3:** Comparison of asexual parasite count per micro-liter (p/μl) and RDT result in health centres and household surveys, Butajira area, Ethiopia, 2008-2010

**Setting/*Plasmodium***	***Plasmodium vivax *(n = 220)**		*Plasmodium falciparum *(n = 85)*	
**Health centres visit**	**RDT negative, n**	**RDT positive, n**	**RDT negative, n**	**RDT positive, n**
60-199	0	0	0	1
200-499	0	5	0	5
500-4,999	3	107	2	19
5,000-90,000	7	98	3	55
Total, n (%)	10 (4.5)	210 (95.5)	5 (5.9)	80 (94.1)
Setting/*Plasmodium*	*Plasmodium vivax* (n = 68)		*Plasmodium falciparum* (n = 13)	
Household surveys	RDT negative, n	RDT positive, n	RDT negative, n	RDT positive, n
40-199	2	3	1	0
200-499	0	4	0	0
500-4,999	4	36	0	6
5,000-90,000	1	18	0	6
Total, n (%)	7 (10.3)	61 (89.7)	1 (7.7)	12 (92.3)

## Discussion

This study found varying precision of CareStart™ RDT to detect *P. falciparum* and *P. vivax* in “active” and clinical settings of highland-fringe areas of south-central Ethiopia. A greater proportion of RDTs than slides gave positive result for *Plasmodium*. In the survey, the test showed low sensitivity and high specificity for *P. falciparum,* but vice versa for *P. vivax*. The test revealed lower PPV for *P. falciparum* and lower NPV for *P. vivax*. Overall, season substantially influences RDT precision with good sensitivity at health facilities compared to household survey. However, specificity was found to be low in both settings with comparable results. Interestingly, better NPV obtained unlike lowest PPV in both survey settings. RDT showed low sensitivity in children aged below 10 years. No strong evidence for contribution of low parasite density for missed *Plasmodium* detection by the test product.

This study has got some limitations. This study used light microscopy for comparison of performance of RDT, in spite of its shortcomings related to variable techniques in specimen preparation and slide reading, and mainly the level of expertise of the examining microscopist [11]. Thus, PCR could be the ideal gold standard in RDT evaluation [11], as applied in recent studies [38,39]. The use of PCR appears plausible in hypoendemic areas like the present study area [40]. There might be a possibility of multiple enrollments of participants between the two survey settings. Some of the strengths of this study include generating large sample data in different seasons at highlands of low malaria endemicity unlike other studies recent studies. RDT evaluation was performed using well trained and competent health personnel at different settings. It is believed that the strengths of this study outweigh the limitations. Thus, conclusions drawn and recommendations suggested are considerably valuable both in improving malaria control efforts and future research endeavor.

The present finding of low sensitivity for *P. falciparum* (87.5%) and *P. vivax* (92.8%) using CareStart™ RDT is comparable with a study conducted in south-west (85.6% for *P. falciparum,* and 85.0% for *P. vivax*) and North-West (92.9% for *P. falciparum*, 90.9% for *P. vivax*) Ethiopian highland areas [21,24]. But the present finding reported lower sensitivity compared to other studies in South (99.4% for *P. falciparum* and *P. vivax*); North-East (98.5% *P. falciparum* and 98.0% for *P. vivax*); and South-West (96.4% for *P. falciparum*, and 95.3% for *P. vivax*) Ethiopia [22,23,25]. The low PPV value obtained in this study is in line with a study in South-West Ethiopia [21], and in low endemicity areas elsewhere [27].

The varying RDT performance in different seasons in the present household survey is generally consistent with another study in hypoendemic highland zones in Kenya and Uganda [26]. Moreover, the finding of low PPV and temporally varying correspondingly with prevalence using household survey is in agreement with other studies [26,40]. However, the results with good sensitivity and specificity from health facility were consistent with other studies done at health facilities [22,23,25]. The present finding of the lowest PPV and NPV during two months, November and December 2009, end of malaria transmission season normally in most parts of Ethiopia. The present result is in line with a recent study [40]. The finding of less variability in RDT performance in different age categories is consistent with an evidence that showed age has no effect on malaria RDT performance [41].

Various factors such as clinical and epidemiologic characteristics of the study populations, reference standards and products of different lots influence the results of RDT-based diagnosis [11,13,16]. Thus, comparison of RDT results difficult. Similarly, the present study used data generated from large population and varying transmission seasons. However, field evaluation of CareStart™ RDT precision used a short duration of peak malaria transmission.

The present finding of low sensitivity of *P. vivax* (health facilities) and *P. falciparum* (survey) disagrees with other studies that consistently found multi-species RDTs to better detect *P. falciparum* infection than non-falciparum infection, most probably *P. vivax*[12,42]. The low sensitivity in health centres for *P. vivax* may be interpreted as due to low performance of multi-species RDTs for this species. The low sensitivity obtained in this study may be explained by several factors pertinent to the manufacturing process and environmental conditions [11,16,20]. The present study was performed in highland area with no extreme weather condition.

In addition, low-prevalence, or hypoendemic, malaria poses particular diagnostic challenges since low population prevalence reduces the PPV of tests [40]. Moreover, test sensitivity suffers when parasite densities within individual infections are low [43]. The hypoendemicity diminishes test sensitivity, as well as the PPV [43]. Very low overall prevalence of malaria was found in the present study area [31], which is known to influence RDT performance as reported [40].

The varying performance of sensitivity for *P. falciparum* and *P. vivax* for survey and health facility might be interpreted as the difference in clinical condition of patients. Study participants visiting health facilities might be clinically more specific than otherwise. Review evidences showed that higher RDT sensitivity in studies involving patients seeking relief from moderate to severe disease in clinical settings while low RDT sensitivity in studies that enrolled patients by means of “active” case finding [11,41].

The RDT had low NPV for *P. vivax* at both survey and health centres. A past study showed that less sensitive for non-*P. falciparum* than for *P. falciparum*[44]*.* There is rich evidence that extremely low sensitivity for both HRP-2 and p-LDH tests and batch specific problems were suspected [16]. This implies that RDT missed some malaria cases or false negatives were higher. Review evidence cited a study that occasional false negative results may be caused by: (i) deletion or mutation of the HRP-2 gene; (ii) anti-HRP-2 antibodies in humans may explain why some tests were negative despite significant parasitemia; and (iii) presence of an inhibitor in the patient’s blood preventing development of the control line [13]. The present finding of low PPV in *P. falciparum* is likely due to sequestration [45]. False positive RDT results occur in a few percent of tests, which can be due to cross-reactivity with rheumatoid factor in blood [16,20]. Cross-reactivity with heterophile antibodies may also occur [13].

## Conclusions

In conclusion, it appears plausible to put malaria RDT quality control system in place for health systems operating in highlands with low endemicity and seasonally varying transmission where *P. falciparum* and *P. vivax* co-exist. CareStart™ RDT could be used for epidemiological studies and results interpreted cautiously by considering its seasonal variation. Future research targeted to RDT evaluation should consider the use of a more sensitive reference standard such as PCR.

## Competing interests

The author(s) declare that they have no competing interests.

## Authors’ contribution

AW contributed to conception and design, data acquisition, analysis and interpretation, and drafting the manuscript. WD substantially contributed to conception and design of the study and reviewing the manuscript, revisiting it critically for important intellectual content. AA substantially contributed to conception and design of the study, reviewing the manuscript, and revisiting it critically for important intellectual content. BL substantially contributed to conception and design, analysis and interpretation of data, reviewing the manuscript, revisiting it critically for important intellectual content. BL, AA, WD and AW reviewed the paper and all authors approved the final version.
